# Effects of Sulla Flexuosa Hay as Alternative Feed Resource on Goat’s Milk Production and Quality

**DOI:** 10.3390/ani13040709

**Published:** 2023-02-17

**Authors:** Soumaya Boukrouh, Ali Noutfia, Nassim Moula, Claire Avril, Jean-Luc Hornick, Mouad Chentouf, Jean-François Cabaraux

**Affiliations:** 1 Department of Veterinary Management of Animal Resources, FARAH Center, Faculty of Veterinary Medicine, University of Liège, 4000 Liège, Belgium; 2 Regional Center of Agricultural Research of Tangier, National Institute of Agricultural Research, Rabat 10090, Morocco; 3 HEPH Condorcet, Agronomy Category, 7800 Ath, Belgium

**Keywords:** *Hedysarum flexuosum*, legume, milk yield, fatty acid, antioxidant

## Abstract

**Simple Summary:**

Sulla flexuosa is a phenolic-rich legume endemic in some southern Mediterranean areas. This plant could be used as an alternative feed resource for goats, but it requires some investigation. Incorporating 35% and 70% DM hay of Sulla flexuosa into the diet did not affect either milk production or physicochemical composition. However, the higher incorporation of 70% in the diet improved the antioxidant capacity and introduced some healthier fatty acids in milk, including docosahexaenoic acid. Therefore, Sulla flexuosa can be used as interesting alternative forage in goat diets.

**Abstract:**

Sulla flexuosa (*Hedysarum flexuosum* L.) is an endemic legume growing in some Mediterranean areas in rainfed and cold mountainous conditions. It could be used in goat diets as an alternative protein source instead of alfalfa to supplement forest rangeland. This study aimed to test the effects of incorporating Sulla flexuosa (SF) hay in the diet of Beni Arouss goats on their milk production and quality. The hay was introduced at two levels, i.e., 35 or 70% (SF70), on a DM basis; it partially or totally replaced the alfalfa hay of the control diet. Sulla flexuosa incorporation did not affect milk production or physicochemical composition. However, milk FA content varied in proportion to the percentage of SF incorporation. The SF70 diet was associated with increased milk levels in C18:1n-9, C18:2n-6, C18:3n-3, and C22:6n-3 and total monounsaturated, polyunsaturated, and n-3 fatty acids. As a consequence, atherogenic and thrombogenic indices were improved. Additionally, better antioxidant capacity was observed in SF70.

## 1. Introduction

In northern Morocco, in rural areas, the population generally lives under precarious conditions. Goat breeding has been the main occupation of many people since ancient times, and goat milk is always a fundamental part of their income, diet, and cultural heritage [1]. In Morocco, goat milk reached 46,953 metric tons in 2018 [2].

Nowadays, consumer interest in food with important nutritional and health value is increasing. Regarding dairy products, the demand for goat milk is rising, especially for its richness in calcium, phosphorus, and vitamins, which are necessary for bone mineralisation [3]. Moreover, goat milk is characterised by low allergenicity due to its better digestibility relative to bovine milk [4]. Furthermore, goat milk presents a composition similar to breast milk, making it a better recommendation for breastfeeding newborns [5].

The goat diet in the Mediterranean region consists mainly of forest pastures [6]. In northern Morocco, due to the overuse of silvopastoral resources by the local population [6] and due to the recurrent drought period [7], rangelands are characterised by limited forage availability and seasonal and annual fluctuations [6]. Thus, farmers have to supplement pastoral resources with conventional concentrates to meet the goats’ nutritional requirements. However, some concentrates compete with human food, present price volatility, and are expensive for the breeders. Using cultivated forage could reduce the amount of used concentrates up to the suitable forage-to-concentrate ratio while ensuring animal productivity, and could alleviate the pressure on the forest to sustain it.

In some Mediterranean areas such as Italy and Spain, the legume Sulla coronaria (*Hedysarum coronarium* L.) is typical of cereal-based crop rotations and is used as fresh forage, hay, and silage and for grazing. It showed interesting results for milk production and cheese and their quality [8,9,10]. In the region of northern Morocco, Sulla flexuosa (*Hedysarum flexuosum* L.) is an endemic legume [11] growing under mountainous rainfed and colder conditions. Few studies about its nutritive value were reported [12]. They showed promising results in crude protein content, adaptability, productivity, and tannic and nontannic phenol content.

To the best of the authors’ knowledge, the effect of Sulla flexuosa on goat milk production and quality has not yet been studied. What about its digestibility, the impacts of its tannins, and its fatty acid profile? This study aimed to analyse the effects of incorporating Sulla flexuosa as forage and as a local alternative feed resource into the goat diet on milk production and quality. Promising results could allow the spreading of this plant in the Mediterranean basin.

## 2. Materials and Methods

### 2.1. Experimental Animals and Diets

The study was conducted at the Regional Agricultural Research Center of Tangier (INRA—Tangier, Morocco), exactly at the Bougdour experimental station (35°67′ N and 5°85′ W). G*Power 3.1.4.9 was used to estimate the sample size (number of goats). Assuming a statistical power of 0.95 and a significance level of *p <* 0.05, the required sample for our study was estimated as “24” goats to expect a statistically significant difference. To remedy any surprise, we decided to analyse thirty lactating goats.

Thirty multiparous local “Beni Arouss” goats were divided into three homogeneous groups based on body weight (38.25 ± 0.50 kg), parity (2.5 ± 0.21), and milk production in the previous lactations (527 ± 63 g/d). In each group, the animals (ten) were housed in the same pen, with metal barriers separating the experimental groups. The thermal conditions and humidity were controlled. Each group was randomly allocated to a treatment. The animals of the control group (Co, *n* = 10) were fed a diet containing alfalfa hay, wheat straw, and barley and oat grains. The tested animals received a diet where the alfalfa hay and the wheat straw were either partially (SF35, *n* = 10) or totally (SF70, *n* = 10) replaced by Sulla flexuosa (SF) hay. The forage-to-concentrate ratio was 70:30; the diets were formulated to meet the requirements of lactating multiparous goats [13] and were isoenergetic and isonitrogenous. The diet transition period began one week before the parturition and lasted for two weeks. Sulla flexuosa was cultivated at the experimental station and cut at the beginning of the flowering stage; it was dried in an open, naturally ventilated building to make hay. The other feedstuffs were bought from the local market. One representative sample of each raw material was collected at the beginning of the experiment and of each diet twice a week during the first three weeks of the trial. The diets, ingredient chemical composition, and diet chemical composition are shown, respectively, in Table 1, Table 2 and Table 3. The forages were minced in a forage chopper with a 2.5 cm screen and mixed manually with the concentrate in the feeder. The goats were not fed individually but in their groups. They were fed twice a day at 0800 h and 1800 h; the amount of diet distributed was adjusted to obtain about 10% refusal. The animals had ad libitum access to fresh and clean water. All the study procedures were approved by the Regional Center of Agricultural Research of Tangier (number: 01/CRRAT/2021).

### 2.2. Feed Analysis

In order to formulate the diets, a representative sample of each feedstuff was collected at the beginning of the experiment. The diets were sampled twice a week during the first three weeks of the trial. All samples were analysed at the INRA-Tangier laboratory. They were dried and ground using a Wiley mill with a 1 mm screen and stored in Kraft bags in a desiccator. The AOAC [14] methods were used for analyses. In brief, DM was determined by drying 100 g of fresh samples in a ventilated oven at 105 ± 1.0 °C until constant weight (method 934.01). Ash content was determined by incinerating 2 g DM in a muffle furnace at 550 °C for 12 h (method 942.05). Ether extract (EE) was extracted using diethyl ether as a solvent in the Soxhlet apparatus (method 963.15). After using the Kjeldahl method (mineralisation, distillation, and titration) (method 977.02), the CP content was determined by multiplying the nitrogen content by 6.25. The crude fiber (CF), ADL, ADF, and NDF contents were analysed using an ANKOM^®^ 200 Fiber Analyser (ANKOM Technology, Macedon, NY, USA); CF was determined according to method 962.09; and ADL, ADF, and NDF were analysed following the method of Van Soest et al. [15]. 

Determination of NDF was performed using α-amylase and sodium sulphite. The nitrogen-free extract (NFE) content was estimated according to the formula (all in g/kg DM):

NFE = 1000 − (EE + CP + CF + Ash)(1)

The in vitro enzymatic dry matter (IVDMD) and organic matter (IVOMD) digestibility were determined using the method of Aufrère and Michalet-Doreau [16] (Supplementary Material S1). The metabolisable energy (ME) of the experimental diets was estimated based on IVDMD (%) according to the formula given by AOAC [14]:

ME (MJ/kg DM) = 0.17 × IVDMD − 2(2)

In vitro enzymatic CP degradability was determined as described by Aufrère and Cartailler [17] (Supplementary Material S2).

The forage unit for milk production (FMU; 1 FMU = 1700 kcal or 7.12 MJ), the digestible proteins in the intestines allowed by nitrogen (PDIN), and the digestible proteins in the intestines allowed by energy (PDIE) were calculated by using the INRAtion^®^ software (INRAE, Paris, France). The rumen protein balance (RuProBal) was obtained according to the formula:

(PDIN − PDIE)/0.64(3)

Quantification of total phenols (TP) and total tannins (TT) was performed according to the procedure described by Makkar et al. [18]. Condensed tannins (CTs) were assayed by Porter et al.’s [19] method (Supplementary Material S3).

The fatty acid (FA) profile of the feedstuffs was extracted using sulfuric acid and was determined by gas chromatography as described by O’Fallon et al. [20] (Supplementary Material S4). The FA profiles of the feedstuffs were used to calculate the FA profiles of the three diets.

### 2.3. Milk Production and Composition

The data collection began one week after the parturition and lasted ten weeks (70 days). Milk production was measured and sampled every Friday. The goats were milked twice, at 0800 h and 1800 h. After mixing the evening and morning milking yields, a 200 mL sample was collected, covered with aluminium foil to protect it from light, and stored at −80 °C before analysis. The suckling kids were separated from their mothers the day before sampling. After the milk sample collection, the rest of the milk was given to kids using clean nursing bottles.

Milk production per lactation was estimated by the Fleischmann method by using the simplified formula of El Otmani et al. [21] (Supplementary Material S5).

Milk’s chemical composition and antioxidant capacity were analysed on each sample (*n* = 80 samples per group); the FA profile was determined on the samples of the odd-numbered weeks (weeks 1, 3, 5, 7, and 9) (*n* = 40 samples per group). Due to the unsynchronised kiddings, some goats could not be sampled at the beginning of the trial.

The lactose, nonfat solids, fat, and protein contents were determined by infrared using MilkoScan™Minor (FOSS, Hilleroed, Denmark). The total solids and ash contents were determined by the AOAC methods [14]. The ash was analysed by incinerating 5 mL of milk in a muffle furnace for 3 h at 550 °C (945.46 method). Total solids were estimated by drying 5 mL of milk in a ventilated oven at 105 ± 1.0 °C until constant weight (925.23 method).

Titratable acidity was determined by 10 mL milk titration using 0.1 N sodium hydroxide, as described by Almeida et al. [22]. Milk pH was measured using a pH meter pen (HANNA HI 98120, Lingolsheim, France). The energy-corrected milk (ECM), fat-corrected milk (FCM), and net energy of milk (NEmilk) were calculated from the daily milk yield and milk composition. The 4% FCM was calculated according to NRC [23] using the equation:

FCM (4%) (g/day) = 0.4 × milk production (g/day) + 15 × fat production (g/day)(4)

The ECM was calculated using the formula described by NRC [23]:

ECM (g/day) = Milk production (g/day) × (0.38 × fat (%) + 0.24 × protein (%) + 0.17 × lactose (%))/3.14(5)

The NEmilk was calculated based on the gross energy per kilogram in fat, protein, and lactose using the NRC equation [23]:

NEmilk (Mcal/kg) = 0.0929 × fat (%) + 0.0563 × protein (%) + 0.0395 × lactose (%) (6)

### 2.4. Milk Antioxidant Capacity

All samples were prepared at room temperature and in low-light conditions. The milk TP compounds were extracted and subsequently quantified using the Folin-Ciocalteu method [24]. The 2,2-diphényl 1-picrylhydrazyle inhibition (DPPH) scavenging activity was quantified according to the method described by Alyaqoubi et al. [25]. The ferric-reducing ability of plasma (FRAP) in milk was determined using the method of Benzie and Strain [26]. All these methods are described in Supplementary Material S6. 

### 2.5. Milk Fatty Acid Profile

The fatty acid profile of milk was also determined by gas chromatography following the same methodology used for the feedstuffs. The observed FAs were grouped into different categories [27] (Supplementary Material S4 and S7).

### 2.6. Statistical Analysis

The experimental data were analysed using the SAS software version 9.4 (SAS Inst. Inc., Cary, NC, USA). The normality of the data was verified. The production, chemical composition, FA proportion, and antioxidant capacity of milk were analysed using the PROC MIXED function, including the random effect of goats, the fixed effects of the diet (Co, SF35, and SF70), the sampling week (j = 10 for production, composition, and antioxidant capacity and j = 5 for fatty acid profile), and their interaction according to the model:

Y_ijk_ = μ + D_i_ + P_j_ +(D×P)_ij_ + G_ijk_ + ε_ijkn_, (7)

where Y_ijk_ is the dependent variable, μ is the general mean, D_i_ is the effect of diet, P_j_ is the effect of the sampling week, (D × P)_ij_ is the interaction between diet and sampling week, G_ijk_ is the goat’s random effect associated to an AR1 covariance structure, and ε_ijkn_ is the random residual effect. The analysis has been performed considering repeated measurements on goats. The CONTRAST statement was used in SAS to estimate “contrasts” values in order to study the linearity of the results. Post hoc analyses were performed using the Tukey test when the results for a parameter were significantly different according to the diets. The significance was set at *p <* 0.05. 

## 3. Results

### 3.1. Diets

The diets were statistically isoenergetic and isoproteinic (Table 3). The phenol and tannin contents increased with the rising incorporation of SF in the diets. The FA profile differed in the three diets. 

### 3.2. Milk Production and Composition

Table 4 shows that the daily milk composition, production, and acidity results were not affected by SF incorporation into the diet. The effect of SF incorporation was nonlinear for all parameters. However, as expected, the effect of the lactation period was significant (*p <* 0.05) on all composition parameters except for lactose, total solids, and ash for the milk yield and fat for milk production. 

### 3.3. Milk Fatty Acid

The individual profiles, summaries, ratios, and indexes of milk FAs according to the diet are given in Table 5. The gradual incorporation of SF into the diets was not systematically correlated to the results; the SF35 diet showed results either intermediate to the extreme diets or similar to one of them. 

Myristic (C14:0), myristoleic (C14:1), pentadecanoic (C15:0), margaric (C17:0), margaroleic (C17:1), stearic (C18:0), oleic (C18:1n-9), linoleic (LA, C18:2n-6), trans-linoleic (6t-C18:2n-6), α-linolenic (ALA, C18:3n-3), arachidic (C20:0), eicosatrienoic (C20:3n-3), dihomo-gamma-linolenic (C20:3n-6), arachidonic (ARA, C20:4n-6), heneicosylic (C21:0), erucic (C22:1n-9), and docosahexaenoic (DHA, C22:6n-3) acids were significantly affected by the diet (*p <* 0.05). The SF70 diet significantly increased C14:1, C18:2n-6, C18:3n-3, C22:1n-9, and C22:6n-3 and decreased C14:0, C17:1, C18:0, and C20:0 proportions in milk fat compared to the Co and SF35 diets. The SF35 and SF70 diets increased C20:3n-3 and C21:0 and decreased C17:0, 6t-C18:2n-6, C20:3n-6, and C20:4n-6 proportions in milk fat compared to the Co diet. The lactation period also had a significant effect on some FAs.

Concerning FA summaries, the SF diet significantly increased LCFA, MUFA, EPA + DHA, and n-9 and decreased SFA proportions compared to the Co diet. In addition, the SF70 diet significantly increased PUFA, n-3, and n-6 proportions compared to the SF35 and Co diets.

For FA ratios, the SF70 diet significantly increased PUFA/SFA and UFA/SFA and decreased MUFA/PUFA compared to the Co and SF35 diets. The SF incorporation in diets did not affect the LA/ALA and n-6/n-3 ratios.

For indexes, the SF70 diet significantly increased Δ9C14 and Δ9C18 activities compared to the two other diets. The experimental diets decreased AI and TI indexes compared to the Co diet, with a higher effect of SF70 for TI.

For linearity, a significant and nonlinear effect of SF hay incorporation level on FA profile was observed for C15:0 and C21:0. Moreover, EPA, DFA, and n-6/n-3 had a nonsignificant but linear effect of SF incorporation.

### 3.4. Milk Antioxidant Activity

For antioxidant activity (Table 6), regarding TP and FRAP, SF70 showed higher values than Co and SF35 diets (*p <* 0.05). Contemporary DPPH values were higher with both SF integrations (SF35 and SF70) than with the Co diet (*p <* 0.05). The effects of SF incorporation were nonlinear.

## 4. Discussion

This study aimed to evaluate SF hay effects on milk production parameters in Beni Arouss goat, a dual-purpose (milk and meat) north Moroccan indigenous breed well known for its rusticity.

### 4.1. Milk Production and Physicochemical Composition

Overall, the daily and total milk yields of the Beni Arouss goat, which is a double-purpose breed (meat and milk), were lower than those of dairy goat breeds but close to those reported in the literature for local Beni Arouss and Draa goats [21,28]. The present results were nevertheless higher than the values reported by El Otmani et al. [21] for the same breed and could be explained by higher dietary energy and protein proportions in this trial. Compared to the control diet, the experimental diets contained condensed tannin (CT) concentrations that were higher but below 3–5% DM. Below this threshold, ruminal activity and feed digestion would not be impaired due to CT-protein binding in the rumen [29]. Indeed, De Lucena et al. [30] reported a general depressive effect in milk yield with high polyphenol concentrations in sheep and goat diets. The SF hay could thus be distributed to goats without such adverse effects. In the present trial, due to diet similarities regarding CP, metabolisable energy, and all types of fibers, close data were observed for milk production and quality in all groups. The observed fat milk percentages (2.16–2.32%) were lower than the values reported in the literature for goats (4.0–4.5%) [31] but similar to those of El Otmani et al. [21] with the same breed but very different diets. It thus seems that a low-fat proportion in milk characterises the Beni Arouss goat breed. The observed protein, lactose, and ash proportions were similar to the averaged values reported by Lad et al. [31] for goats. As expected, the milk composition varied according to the lactation stage. Thus, the hay of SF did not negatively affect milk composition and production in Beni Arouss goats.

### 4.2. Milk Fatty Acids 

Unlike milk production and composition, the milk fatty acid profile was significantly affected by the SF incorporation.

Milk C14:0 is synthesised de novo in the mammary gland and milk C14:1 originates exclusively from the desaturation of C14:0 at this site [32]. The Δ9C14 ratio is considered the marker for delta-9 desaturase activity in the udder [30]. The lower C14:1 proportion observed with the Co and SF35 diets was confirmed by the lower Δ9C14 ratio observed in the Co and SF35 groups compared to SF70, with, respectively, 0.045 vs. 0.061. Similar to the present results, Purba et al. [33] and Buccioni et al. [34] suggested that dietary tannins can increase the expression of Δ9 desaturase activity, as observed in the SF70 group. Despite the lack of significant difference between the SF35 and Co groups, the significant and linear effect of the level of SF incorporation suggests that SF could have a dose-dependent impact. 

The present results showed a lower C17:0 proportion for the SF35 and SF70 compared to the Co diet (1.08 vs. 1.55 g/100 g FA, respectively, for the SF35 and SF70 diets and the Co diet). The ruminal microorganisms synthesise C17:0 by elongation of propionate. Cabiddu et al. [35] reported an increase in C17:0 percentage in the milk of sheep receiving a PolyEthylen Glycol supplementation in the diet, and this molecule has the ability to neutralise tannins. This lower C17:0 proportion in milk could be due to a ruminal microbiota modification, impairing the saturation process in the rumen [36].

There was a smaller C18:0 milk proportion with SF70 (8.06 vs. 12.55 g/100 g FA, respectively, for the SF70 diets and both the Co and SF35 diets). The observation could be explained by different hypotheses: the lower C18:0 proportion in the SF70 diet and the modification of the ruminal microbiota and, thus, biohydrogenation (as for C17:0) and higher desaturation of C18:0 in C18:1 in the mammary gland as shown by the higher Δ9C18 activity.

The modification of the ruminal biohydrogenation process could also explain the higher C18:2n-6 and C18:3n-3 proportion in SF70 milk [34]. Usually, around 90% of C18:3n-3 and C18:2n-6 are biohydrogenated in the rumen [37]. These two FAs represented 51%, 57%, and 69% of the observed PUFA proportion, respectively, for the Co, SF35, and SF70 diets. The highest level of SF was thus required to observe an effect. The C20:3n-6 and C20:4n-6 FAs result from the C18:2n-6 elongations and desaturations. A lower proportion—not SF dose-dependent—of these two FAs in the SF35 and SF70 milk, despite a higher C18:2n-6 proportion in SF70 milk, is hard to explain. Sulla flexuosa could limit the activities of some enzymes. The capacity of conversion of C18:3n-3 to health-promoting n-3 long-chain PUFA (eicosapentaenoic acid (EPA, 20:5n-3) and docosahexaenoic acid (DHA, 22:6n-3)) is limited in human metabolism, which strengthens its dietary supply’s importance. Like C18:2n-6, the C18:3n-3 milk proportion was greater in the SF70 group than in the two other groups (respectively, 2.73 vs. 1.39 g/100g FA). Although the milk concentration of DHA was also significantly higher in the SF70 group, the EPA milk proportions did not differ between the three groups. An absence of a parallel increase in n-3 and n-6 LCFA between groups was thus observed; tannins’ impact on enzyme competition between n-3 et n-6 metabolism is not currently understood. 

### 4.3. Fatty Acid Summaries, Ratios, and Indexes 

The calculated FA summaries, ratios, and indexes of a foodstuff are usually considered indicators to the consumer for choosing a healthy diet. Therefore, long-chain n-3 and n-6 PUFA are considered bioregulators of important cellular processes and are associated with immune system functionality and development. The MUFAs improve cardiovascular health by decreasing inflammation and reducing total and low-density lipoprotein (LDL)-cholesterol. The SFAs have negative consequences on human health by increasing the danger of cardiovascular disorders and the level of blood plasma cholesterol [38]. In the present trial, on average, the PUFA and MUFA proportions observed in the three groups were higher, and consequently, the SFA proportion was lower than the values reported for goat milk, with, respectively, 3.7, 24.5, and 68.8% [39]. The increased proportion of SF in the diets increased MUFA and decreased SFA proportions in milk. The significantly greater PUFA level with SF70 was because of the higher C18:2n-6 and C18:3n-3 levels (and cumulative overall higher n-3 FA). The difference in the MUFA milk proportions according to the group was mainly caused by the differences observed in the C18:1n-9 proportions; this last one has beneficial effects on human health [38]. Similar to the current study, a greater unsaturated fatty acid (UFA) proportion and a lower SFA proportion in milk from goats and ewes supplemented with tannin-rich diets were reported and explained by a change in the ruminal microbial population activity manifested by a decrease in FA biohydrogenation [30].

In goat milk, the PUFA/SFA ratio ranges from 0.04 to 0.18, the n-6/n-3 ratio from 1.49 to 6.60, the AI ratio from 1.89 to 2.77, the TI ratio from 2.04 to 3.20, and the LA/ALA ratio from 1.15 to 10.67 [21,38,40,41]. The PUFA/SFA and LA/ALA ratios detected in our study were in the range, whereas the other indicators were lower. The incorporation of 70% SF significantly enhanced the PUFA/SFA ratio and the AI and TI indexes. De Lucena et al. [30] reported decreased AI and TI in Saanen goat milk with up to 28 g tannins per kg DM diet; the SF70 diet contained 15.2 g/kg DM. 

The SF incorporation in diets did not affect the n-6/n-3 ratio. The recommended ratio ranges around 1-2:1 [42], which we observed in the three groups. Some authors reported ratios up to 6.6 in goat milk (see above). An optimal n-6/n-3 ratio could have some anti-inflammatory and anticarcinogenic effects. On the other hand, LA and n-6 FA could protect against cardiovascular diseases [43]. Some authors [38] thus question the usefulness of some of these indicators, arguing that they do not reflect the actual effect of each FA present in a FA group or an indicator. For example, all n-3 FAs do not positively impact human/animal metabolism in the same way. In the present trial, the most interesting FAs, such as C18:1n-9, C18:2n-6, C18:3n-3, and DHA, were observed in higher concentrations in SF70 milk.

### 4.4. Antioxidant Activity

It is well known that animals at early lactation may face a mobilisation of lipids from their adipose tissue and produce high amounts of reactive oxygen species, which requires a more intensive antioxidant defence of the body [44]. The SF70 milk presented the highest TP content, which is explained by this diet’s highest TP content and its transfer to the milk via the bloodstream [8]. The TP, playing an antioxidant role, and FRAP and DPPH, which measure the antioxidant activity [45], showed the highest values in SF70 milk. Di Trana et al. [8] also reported a positive correlation between goat plasma antioxidant capacity and Sulla coronaria CT intake. 

DPPH values in the present study were lower than the values of 71% reported by Lakram et al. [46] for the effects of detoxified *Argania spinosa* pressed cake on antioxidant activity in Alpine goat milk. Alyakoubi et al. [25] also reported higher values but probably due to their extraction method with HCl instead of direct analysis. Other authors [47,48] also reported values (18–36%) close to the ones observed in the present trial, with either jujube pulp or purple corn (Zea mays L.) rich in anthocyanins as the antioxidant. For FRAP activity, the observed values (0.760–1.103 mmol FeSO_4_/L) were higher than 0.19 mmol FeSO_4_/L considered as the critical value to limit oxidative damage to milk [49]. The antioxidant activity in the SF35 milk was different for these two parameters, not significantly different from SF70 for DPPH, and not significantly different from Co for FRAP; it was probably due to the used methods. It is thus important to analyse DPPH and FRAP activities in order to well qualify the antioxidant activity.

The higher proportions of PUFA, MUFA, n-3, n-6, and n-9 in SF70 milk could, therefore, also be due to the protection of the UFA against biohydrogenation and/or oxidation because of the higher TP and TC contents in milk and diet. It was, however, surprising to observe the nonlinearity of the effect of SF diet incorporation on these previous parameters. 

## 5. Conclusions

Sulla flexuosa hay in the diet of dairy goats had no adverse effects on animal production and raw milk characteristics. Tannins present in Sulla flexuosa hay probably had a protective effect on fatty acid biohydrogenation in the rumen and an impact on fatty acid desaturating enzymes in the mammary gland. The data of this study suggest that Sulla flexuosa hay should be incorporated with no less than half of dry matter intake to show noticeable effects. Therefore, Sulla flexuosa should be suggested as an available alternative forage and protein resource in the lactating goat diet. Further studies are recommended to test its use as fresh or silage forage and to explore its effect on ruminal microbiota and different goat milk product characteristics.

## Figures and Tables

**Table 1:** Diet composition.

	Co	SF35	SF70
Diet ingredients (on DM basis)			
Sulla hay (g/kg DM)	0	350	700
Alfalfa hay (g/kg DM)	450	225	0
Wheat straw (g/kg DM)	250	125	0
Barley grains (g/kg DM)	137	137	137
Oat grains (g/kg DM)	140	140	140
Vitamin-mineral Supplement * (g/kg DM)	23	23	23

* Per kg vitamin-mineral supplement: 350,000 IU retinol, 80,000 IU cholecalciferol, 1500 mg α-tocopherol, 4 g Manganese, 5 g Zinc, 240 g Calcium, 50 g Magnesium.

**Table 2:** Chemical composition of the diet ingredients.

	Sulla Hay	Alfalfa Hay	Wheat Straw	Oats Grains	Barley Grains
Dry matter (DM; g/kg)	907	911	880	917	903
Ash (g/kg DM)	130	102	74.7	29.6	28.1
Crude protein (g/kg DM)	162	215	27.8	118	88.7
Ether extract (g/kg DM)	33.2	39.5	10.5	33.5	79.8
Neutral detergent fiber (g/kg DM)	520	394	800	121	181
Acid detergent fiber (g/kg DM)	412	263	506	94.8	136
Acid detergent lignin (g/kg DM)	118	76.3	150	27.1	38.2
Crude fiber (g/kg DM)	265	202	420	42.6	67.8
Nitrogen-free extract (g/kg DM)	386	441	467	776	736
IVOMD (g/kg DM)	563	676	220	843	803
Metabolisable energy (MJ/kg DM)	7.44	9.61	2.22	11.2	11.9
FMU (unit/kg DM)	0.67	0.82	0.42	1.05	1.07
PDIE (g/kg DM)	93	142	46	85	73
RuProBal	19	22	−41	−17	−27
Total phenols (g/kg DM)	45.3	13.3	0.20	28.8	21.7
Hydrolysable tannins (g/kg DM)	16.9	0.98	0.17	3.2	1.26
Condensed tannins (g/kg DM)	17.5	1.31	0.15	4.01	1.16

IVOMD: in vitro organic matter digestibility; FMU: forage unit for milk production; PDIE: digestible proteins in the intestines allowed by energy; RuProBal: rumen protein balance.

**Table 3:** Chemical composition of the offered diets (*n* = 6 for each group).

	Co	SF35	SF70	SEM	*p*-Value
Nutrient composition					
Dry matter (DM; g/kg)	904	901	900	1.043	0.597
Ash (g/kg DM)	87.2 ^c^	103.8 ^b^	112.1 ^a^	0.362	<0.001
Crude protein (g/kg DM)	159	155	167	0.341	0.101
Ether extract (g/kg DM)	42.3	42.2	44.3	0.075	0.216
Neutral detergent fiber (g/kg DM)	491	489	479	1.621	0.356
Acid detergent fiber (g/kg DM)	331	346	365	2.534	0.486
Acid detergent lignin (g/kg DM)	97.5	103	106	0.255	0.250
Crude fiber (g/kg DM)	30.5	29.8	29.8	0.146	0.583
Nitrogen-free extract (g/kg DM)	207	225	197	0.313	0.112
Metabolisable energy (MJ/kg DM)	9.60	9.50	9.80	0.049	0.190
FMU (unit/kg DM)	0.90	0.90	0.89	0.009	0.070
PDIE (g/kg DM)	96.0	89.9	102	0.145	0.280
PDIN (g/kg DM)	101	104	108	0.264	0.110
RuProBal	−6	0	7		-
Total Phenols (g/kg DM)	15.8 ^c^	25.6 ^b^	40.5 ^a^	0.467	<0.001
Hydrolysable tannins (g/kg DM)	1.09 ^c^	5.94 ^b^	12.4 ^a^	0.109	<0.001
Condensed tannins (g/kg DM)	1.53 ^c^	8.39 ^b^	15.2 ^a^	0.224	<0.001
Fatty acid composition of the diet (g/100g FA) (calculated from feedstuff fatty acid profile)					
C4:0	0.044	0.102	0.154		
C10:0	0.028	0.053	0.073		
C12:0	0.633	0.432	0.248		
C14:0	1.432	0.895	0.404		
C14:1	0.476	0.255	0.054		
C15:0	1.102	0.686	0.307		
C16:0	19.911	19.095	18.360		
C16:1	1.478	0.857	0.290		
C17:0	0.574	0.333	0.112		
C17:1	0.046	0.041	0.037		
C18:0	5.963	3.990	2.191		
9t-C18:1	0.346	0.199	0.065		
C18:1n-9	12.629	12.093	11.611		
C18:2n-6	30.060	27.798	25.746		
C18:3n-3	17.620	28.203	37.883		
C18:3n-6	0.351	0.251	0.159		
C20:0	0.921	0.633	0.371		
C20:1	0.538	0.354	0.187		
C20:2	0.388	0.264	0.127		
C20:3n-3	0.471	0.264	0.076		
C20:4n-6	0.333	0.238	0.127		
C20:5n-3	0.499	0.344	0.203		
C22:0	1.718	1.058	0.456		
C22:1n-9	0.416	0.344	0.253		
C24:0	1.496	0.899	0.355		
C24:1	0.527	0.317	0.152		

Co: control diet; SF35: diet with 35% Sulla flexuosa hay; SF70: diet with 70% Sulla flexuosa hay; FMU: forage unit for milk production; PDIE: digestible proteins in the intestines allowed by energy; RuProBal: rumen protein balance. ^a,b,c^: values followed by different letters in the same row differ tatistically by Tukey’s test at (*p* < 0.05).

**Table 4:** Least square means of production, composition, acidity, and net energy of goat milk according to diet (*n* = 80 for each group).

	Co	SF35	SF70	SEM	*p*-Value			
					Linear	Diet	Week	Diet × Week
Milk yield (kg/lactation)	62.2	64.2	68.1	0.202		0.788	-	-
Chemical composition (%)								
Fat	2.24	2.32	2.16	0.101	0.792	0.876	0.006	0.855
Proteins	3.46	3.30	3.42	0.079	0.866	0.781	<0.001	0.218
Lactose	5.11	5.14	5.06	0.126	0.850	0.960	0.116	0.034
Ash	0.840	0.836	0.775	0.003	0.306	0.547	0.089	0.830
Total solids	11.7	11.6	11.9	0.147	0.636	0.648	0.300	0.033
Milk production (g/day)								
Milk	663	654	590	17.5	0.474	0.742	<0.001	0.054
Energy-corrected milk	532	497	456	0.083	0.296	0.667	0.003	0.094
Fat-corrected milk	494	472	419	0.021	0.376	0.664	<0.001	0.033
Fat	15.3	14.0	12.2	0.224	0.356	0.656	0.073	0.100
Proteins	21.5	19.5	17.9	0.092	0.226	0.474	0.003	0.050
Lactose	34.1	34.7	31.7	0.208	0.675	0.865	<0.001	0.193
Total solids	77.3	74.6	60.6	0.301	0.361	0.428	<0.001	0.050
Ash	5.21	5.14	4.36	0.043	0.351	0.594	0.029	0.306
Net energy of milk (MJ/kg)	2.49	2.49	2.45	0.034	0.817	0.959	<0.001	0.409
pH	6.44	6.50	6.48	0.033	0.615	0.722	0.148	0.200
Acidity (°D)	16.8	15.7	15.8	0.021	0.161	0.216	<0.001	0.067

**Table 5:** Least square means and standard error of the mean of fatty acid profile (g/100 g FA) and summaries (g/100 g FA), ratios, and indexes in goat milk fat according to diet (*n* = 40 for each group).

	Co	SF35	SF70	SEM	*p*-Value			
					Linear	Diet	Week	Diet × Week
Fatty acid profile								
C4:0	1.41	1.41	1.19	0.048	0.108	0.218	0.682	0.308
C6:0	2.11	1.95	1.99	0.074	0.606	0.742	0.277	0.232
C8:0	1.60	1.46	1.29	0.069	0.154	0.359	0.009	0.839
C10:0	5.83	5.37	5.92	0.125	0.821	0.330	0.084	0.456
C11:0	0.183	0.148	0.146	0.008	0.081	0.119	0.022	0.497
C12:0	2.56	2.74	2.38	0.093	0.462	0.501	0.251	0.083
C13:0	0.117	0.136	0.094	0.006	0.223	0.111	0.852	0.994
C14:0	9.52 ^a^	8.29 ^ab^	7.67 ^b^	0.186	0.007	0.020	0.416	0.498
C15:0	1.69 ^ab^	2.14 ^a^	1.41 ^b^	0.097	0.318	0.044	0.177	0.477
C16:0	21.2	21.0	22.3	0.221	0.159	0.211	0.335	0.030
C17:0	1.55 ^a^	1.10 ^b^	1.06 ^b^	0.064	0.006	0.006	0.366	0.466
C18:0	12.8 ^a^	12.3 ^a^	8.06 ^b^	0.289	<0.001	<0.001	0.179	0.112
C20:0	0.432 ^a^	0.433 ^a^	0.305 ^b^	0.016	0.004	0.007	0.708	0.476
C21:0	0.315 ^b^	0.423 ^a^	0.343 ^b^	0.012	0.395	0.005	0.963	0.775
C22:0	0.372	0.370	0.399	0.005	0.046	0.075	0.253	0.887
C23:0	0.131	0.136	0.129	0.002	0.735	0.572	0.838	0.384
C24:0	0.071	0.062	0.058	0.004	0.268	0.513	0.482	0.876
Total SFA	62.0 ^a^	59.4 ^b^	54.5 ^c^	0.408	<0.001	<0.001	0.343	0.336
C14:1	0.365 ^b^	0.410 ^b^	0.481 ^a^	0.012	<0.001	0.002	0.019	0.495
C15:1	1.32	1.53	1.44	0.067	0.582	0.569	0.230	0.022
C16:1	1.03	1.02	1.13	0.034	0.350	0.556	0.827	0.359
C17:1	1.56 ^a^	1.74 ^a^	1.12 ^b^	0.061	0.010	0.003	0.693	0.588
9t-C18:1	0.288	0.323	0.243	0.010	0.236	0.121	0.065	0.380
C18:1n-9	26.3 ^c^	28.7 ^b^	31.6 ^a^	0.342	<0.001	<0.001	0.014	0.187
C20:1	0.223	0.227	0.202	0.011	0.593	0.805	0.805	0.847
C22:1n-9	0.447 ^b^	0.431 ^b^	0.657 ^a^	0.020	<0.001	<0.001	0.741	0.474
C24:1	0.313	0.337	0.326	0.008	0.569	0.564	0.146	0.538
Total MUFA	31.8 ^b^	34.7 ^a^	37.1 ^a^	0.345	<0.001	<0.001	0.018	0.318
6t-C18:2	0.786 ^a^	0.392 ^b^	0.336 ^b^	0.067	0.012	0.017	0.150	0.050
C18:2n-6	1.81 ^b^	1.91 ^b^	2.85 ^a^	0.094	<0.001	0.001	0.240	0.059
C18:3n-3	1.34 ^b^	1.44 ^b^	2.73 ^a^	0.080	<0.001	<0.001	0.002	0.060
C18:3n-6	0.232	0.238	0.179	0.012	0.183	0.292	0.991	0.283
C20:2	0.403	0.380	0.393	0.011	0.752	0.743	0.442	0.473
C20:3n-3	0.255 ^b^	0.328 ^a^	0.372 ^a^	0.011	<0.001	<0.001	0.151	0.117
C20:3n-6	0.465 ^a^	0.350 ^b^	0.356 ^b^	0.011	<0.001	<0.001	0.090	0.202
C20:4n-6 (ARA)	0.334 ^a^	0.203 ^b^	0.202 ^b^	0.009	<0.001	<0.001	0.982	0.996
C20:5n-3 (EPA)	0.360	0.412	0.461	0.012	0.019	0.060	0.934	0.752
C22:2	0.188	0.175	0.230	0.007	0.072	0.068	0.734	0.630
C22:6n-3 (DHA)	0.054 ^b^	0.048 ^b^	0.075 ^a^	0.002	<0.001	<0.001	0.183	0.182
Total PUFA	6.19 ^b^	5.87 ^b^	8.10 ^a^	0.155	<0.001	<0.001	0.054	0.363
Summaries								
SCFA	10.4	10.2	10.4	0.165	0.260	0.284	0.233	0.261
MCFA	12.8 ^a^	11.7 ^ab^	10.8 ^b^	0.205	0.008	0.029	0.291	0.471
LCFA	76.2 ^b^	78.0 ^a^	78.7^a^	0.265	0.007	0.019	0.812	0.266
DFA	50.8	52.8	53.3	0.310	0.032	0.069	0.517	0.771
EPA + DHA	0.414 ^b^	0.461 ^a^	0.537 ^a^	0.012	0.006	0.023	0.968	0.840
n-3	2.01 ^b^	2.23 ^b^	3.64 ^a^	0.089	<0.001	<0.001	0.003	0.092
n-6	2.84 ^b^	2.70 ^b^	3.59 ^a^	0.094	0.013	0.011	0.166	0.061
n-9	27.0 ^c^	29.5 ^b^	32.5 ^a^	0.347	<0.001	<0.001	0.012	0.162
Ratios								
PUFA/SFA	0.100 ^b^	0.099 ^b^	0.149 ^a^	0.003	<0.001	<0.001	0.152	0.292
MUFA/PUFA	5.51 ^a^	6.12 ^a^	4.69 ^b^	0.135	0.038	0.004	0.004	0.698
UFA/SFA	0.619 ^b^	0.687 ^b^	0.838 ^a^	0.012	<0.001	<0.001	0.444	0.428
LA/ALA	1.40	1.53	1.26	0.073	0.558	0.571	0.420	0.016
n-6/n-3	1.44	1.29	1.10	0.129	0.036	0.109	0.528	0.016
Indexes								
AI	1.65 ^a^	1.41 ^b^	1.23 ^b^	0.030	<0.001	<0.001	0.281	0.310
TI	1.83 ^a^	1.59 ^b^	1.19 ^c^	0.032	<0.001	<0.001	0.431	0.051
(C18:0 + C18:1)/C16:0	1.88	1.99	1.82	0.030	0.608	0.305	0.339	0.047
Δ9C14	0.040 ^b^	0.050 ^b^	0.061 ^a^	0.002	<0.001	<0.001	0.094	0.347
Δ9C16	0.046	0.046	0.048	0.002	0.564	0.837	0.840	0.286
Δ9C18	0.674 ^b^	0.703 ^b^	0.799 ^a^	0.007	<0.001	<0.001	0.039	0.061

Co: control diet; SF35: diet with 35% DM Sulla flexuosa hay; SF70: diet with 70% DM Sulla flexuosa hay; SCFA: short-chain fatty acids; MCFA: medium-chain fatty acids; LCFA: long-chain fatty acids; MUFA: mono-unsaturated fatty acids; PUFA: polyunsaturated fatty acids; SFA: saturated fatty acids; DFA: desirable fatty acids; EPA: eicosapentaenoic acid (20:5n-3); DHA: docosahexaenoic (C22:6n-3); UFA: unsaturated fatty acids; AI: atherogenicity index; TI: thrombogenic index; Δ9C14 = (C14:1)/(C14:1 + C14:0) activity of Δ9 desaturase enzyme to convert C14:0 into C14:1; Δ9C16 = (C16:1)/(C16:1 + C16:0) activity of Δ9 desaturase enzyme to convert C16:0 into C16:1; Δ9C18 = (C18:1)/(C18:1 + C18:0) activity of Δ9 desaturase enzyme to convert C18:0 into C18:1; ^a^,^b^,^c^: values followed by different letters in the same row differ statistically by Tukey’s test at (*p <* 0.05).

**Table 6:** Least square means and standard error of the mean of total phenols and antioxidant activities of goat milk according to diet (*n* = 80 for each group).

	Co	SF35	SF70	SEM	*p*-Value			
					Linear	Diet	Week	Diet × Week
Total phenols (mg GAE/L)	29.5 ^b^	30.9 ^b^	39.8 ^a^	0.320	0.111	<0.001	0.030	0.064
FRAP (mmol FeSO_4_/L)	0.758 ^b^	0.762 ^b^	1.103 ^a^	0.002	0.164	<0.001	0.499	0.466
DPPH (%)	30.1 ^b^	34.5 ^a^	35.6 ^a^	0.600	0.129	0.003	0.106	0.025

Co: control diet; SF35: diet with 35% Sulla flexuosa hay; SF70: diet with 70% Sulla flexuosa hay; FRAP: ferric-reducing ability of plasma; GAE: tannic acid equivalent; DPPH: 2,2-diphényl 1-picrylhydrazyle inhibition. ^a,b^: values followed by different letters in the same row differ tatistically by Tukey’s test at (*p* < 0.05).

## Data Availability

The data that support the findings of this study are available upon request from the authors.

## Supplementary Materials

The following are available online at https://www.mdpi.com/article/10.3390/ani13040709/s1, Supplementary material S1: The in vitro enzymatic dry matter (IVDMD) and organic matter (IVOMD) digestibility using the method of Aufrère and Michalet-Doreau [16], Supplementary Material S2: In vitro enzymatic CP degradability using the method of Aufrère and Cartailler [17], Supplementary Material S3: Quantification of total phenols (TPs) and total tannins (TTs) according to the procedure described by Makkar et al. [18] and Condensed tannins (CTs) by Porter et al.’s [19], Supplementary Material S4: The fatty acid (FA) profile of milk and feedstuffs as described by O’Fallon et al. [20], Supplementary Material S5: Milk production per lactation estimation by the Fleischmann method [21], Supplementary Material S6: The milk TP compounds quantification using the Folin-Ciocalteu method [24], the 2,2-diphényl 1-picrylhydrazyle inhibition (DPPH) scavenging activity quantification according to the method of Alyaqoubi et al. [25], the ferric-reducing ability of plasma (FRAP) in milk determination using the method of Benzie and Strain [26], Supplementary Material S7: The observed FAs were grouped into different categories [27,50].
