# Supplementary material for: Effects of Sulla Flexuosa Hay as Alternative Feed Resource on Goat’s Milk Production and Quality
Source: Animals (Basel). 2023 Feb 17;13(4):709. doi: 10.3390/ani13040709 (PMC9952730; doi:10.3390/ani13040709)
Supplement: Supplementary file 1 [file animals-13-00709-s001.zip › animals-2186182-SI.pdf]

Animals - **Supplementary Materials**

Effects of Sulla Flexuosa Hay as Alternative Feed Resource on Goat's Milk Production and Quality

Soumaya Boukrouh [1,2,*], Ali Noutfia [2], Nassim Moula [1], Claire Avril [3], Jean-Luc Hornick [1], Mouad Chentouf [2] and Jean-François Cabaraux [1]

[1] Department of Veterinary Management of Animal Resources, FARAH Center, Faculty of Veterinary Medicine, University of Liège, 4000 Liège, Belgium; nassim.moula@uliege.be (N.M.); jlhornick@uliege.be (J.-L.H.); jfcabaraux@uliege.be (J.-F.C.)

[2] Regional Center of Agricultural Research of Tangier, National Institute of Agricultural Research, 10090 Rabat, Morocco; ali.noutfia@inra.ma (A.N.); mouad.chentouf@inra.ma (M.C.)

[3] HEPH Condorcet, Agronomy Category, 7800 Ath, Belgium; claire.avril@condorcet.be

* Correspondence: s.boukrouh@uliege.be

Animals - **Supplementary Materials**

Supplementary Material S1

In vitro enzymatic dry matter (IVDMD) and organic matter (IVOMD) digestibility

The in vitro enzymatic dry matter (IVDMD) and organic matter (IVOMD) digestibility was determined using the method of Aufrère and Michalet-Doreau [1]. In brief, 0.5 g of dried sample was incubated at 40°C for 24h with 20 mL of a 2% pepsin solution diluted in 0.1 N hydrochloric acid. Then, the sample was solubilized in 50 mL of a buffer solution containing 1 g.l-1 cellulase, shaken and incubated at 40°C for 24 h. After incubation, the sample was rinsed with hot distilled water and then placed in an oven at 60°C until constant weight. It was weighed to determine the IVDMD. The sample was incinerated in the muffle furnace at 550°C for 12 hours to determine IVOMD.

Supplementary Material S2

In vitro enzymatic CP degradability

In vitro enzymatic CP degradability was determined by enzymatic hydrolysis for 1 h by protease extracted from Streptomyces griseus in a borate phosphate buffer at pH 8 as described by (Aufrère and Cartailler [2]). Briefly, in a centrifuge tube, 0.5 g of the sample was mixed with 25 mL of a protease solution and was incubated at 40°C for one hour. The sample was centrifuged for 5 minutes at 3500 rpm and then filtered. The liquid phase was mineralised for one hour at 120°C and for 2 hours at 350°C using sulfuric acid. The tube containing the mineralised sample was connected to the distiller. Finally, the contents of the Erlenmeyer flask were titrated with 0.1 N HCL to determine the digested nitrogenous matter.

Supplementary Material S3

Total phenols and tannins

Quantification of total phenols (TP) and total tannins (TT) was performed according to the procedure described by Makkar *et al.* [3]. Briefly, the extraction was done by mixing 200 mg of finely ground dry sample with 10 mL of 70% acetone in a 25 mL glass beaker. The beaker was subject to ultrasonic treatment for 20 minutes at room temperature, and then the content of the beaker was transferred to centrifuge tubes and subjected to centrifugation for 10 minutes at 3500 rpm at 4°C. In a tube, 0.05 mL of the extract was mixed with 0.5 mL of distilled water, 0.25 mL of Folin Ciocalteu 1N reagent, and 1.25 of 20% sodium carbonate solution. The solution was then vortexed and left in the dark for 40 minutes; the absorbance was read at 725 nm to determine total phenols. In a centrifuge tube, 300 mg of polyvinylpolypyrrolidone was mixed with 3 mL of demineralised water and 3 mL of the extract. The tube was vortexed, kept at 4°C for 15 min, centrifuged at 4°C at 3500 rpm for 10 minutes, and then read at 725 nm to determine non-tannic phenols. Total tannins were calculated as the difference between total phenols and non-tannic phenols. Condensed tannins (CT) were assayed by Porter et al.

[4] method. Briefly, in a glass tube, 0.5 mL of the extract was mixed with 3 mL of Butanol-HCl reagent and 0.1 mL of ferric reagent. The tubes were vortexed and put in a water bath at 97°C for one hour. After cooling, the absorbance was read at 550 nm. The difference between TT and CT deduced hydrolysable tannins (HT).

Supplementary Material S4

Dietary fat extraction and methylesters preparation

Fatty acid profile in feed samples was determined according to the method of O'Fallon, *et al.* [5]. Samples of ground feeds (0.5 g) were mixed with 0.35 ml of 10 (N) KOH and 2.65 ml of methanol. The tubes containing these solutions were incubated at 55 °C for 1.5 h with vigorous hand shaking. Then, 0.29 ml of 24 (N) H_2SO_4 was added. The tube was mixed thoroughly and incubated again at 55 °C for 1.5 h. After FA methyl ester (FAME) synthesis, the tube was cooled in a cold tap water bath, 1.5 ml of hexane was added, and the tube was mixed for 5 min on a vortex. The tubes were centrifuged for 5 min at 2500 rpm and the hexane layer containing FAME was transferred into a vial. The vial was capped and placed at −20 °C until analysis.

Milk fat extraction and methylesters preparation

Milk fat extraction and methylesters preparation was processed according to Barbano et al. [6]. Briefly, milk fat was obtained by a sequence of 3 successive extractions from 10 mL of milk. The first extraction was the addition of 10 mL of 95% alcohol, 25 mL of ethyl ether and 25 mL of petroleum ether, then the decanting of the ether layer. The second extraction was the addition of 5 mL of 95% alcohol, and 15 mL of ethyl and petroleum ether. The third extraction was the addition of only 15 mL of ethyl and petroleum ether. The three solutions were combined, dried and put in hexane. The FA methyl esters (FAMEs) were prepared using 0.5 mL of sodium methoxide in methanol 0.5 N and 1 mL of hexane. The vial was capped and placed at −20 °C until analysis.

Gas-chromatographic procedure the separation and the identification of the FAME from feeds and milk

The fatty acid (FA) profile of the feedstuffs was determined by gas chromatography (GC) with a Varian GC CP 3800 device (Agilent Technologies, Santa Clara, CA, USA) equipped with a split/split less injector, flame ionisation detector, fitted with a capillary column type CP- SIL88 (100m×0.25mm×0.2µm) and helium carrier gas at a flow of 1 mL/min. Before injection, the FA methyl esters were prepared as methyl esters described by AOAC [7] (method 969.33). The oven temperature in the GC was programmed at 50°C and held for 4 min, then increased to 120°C at 10°C/min, held for 1 min, then increased up to 180°C at 5°C/min, held for 18 min, then increased up to 200°C at 2°C/min, and finally increased up to 230°C at 2°C/min and held for 19 min. The injector and detector temperatures were at 270°C and 300°C, respectively. The FA were identified by comparing their retention times with a standard analytical mixture of C4 to C24 FA (Sigma-Aldrich, Darmstadt, Germany), referring to 37 FA.

Supplementary Material S5

Milk production per lactation was estimated by the Fleischmann method by using the

simplified formula of El Otmani *et al.* [8]:

$$Milk\ yield\ = I_1 \times X_1 + \sum_{i=2}^{n}(I_i - I_{i-1}) \times \frac{\mathrm{X}_i - \mathrm{X}_{i-1}}{2} + I_m \times I_n$$

Where:

I_1: number of days between parturition and the first milk recording;

X_1: milk quantity produced at the first milk recording;

I_i: number of days between the parturition and the i milk recording (i = 2,...,n);

X_i: milk quantity produced at the i milk recording (i = 2,…,n);

I_m: number of days between the last milk recording and dry-off date;

X_n: milk quantity produced at the last milk recording;

n = 10.

Supplementary Material S6

Milk total phenolic compounds

The milk TP compounds were extracted and subsequently quantified using the Folin-Ciocalteu method [9]. Briefly, after defrosting the samples in a water bath at 35–40°C, they were homogenized by vortexing for 1 min. Then, in a 25 mL volumetric flask, 8 mL of milk were mixed with 10 mL of methanol–water (1:1, v/v), 500 µl of Carrez I solution containing potassium hexacyanoferrate (II) trihydrate, 500 µl of Carrez II solution and 5 mL of acetonitrile. Following the addition of each solution, the mixture was vortexed. The solution was complemented to 25 mL with methanol–water (1:1, v/v) and the mixture was then allowed to stand for 25 min. The resulting solution was centrifuged at 7800 rpm at 5 C for 15 min. Total phenol compounds in the liquid extract were subsequently quantified using the Folin–Ciocalteu method [9]. The 100 µL of methanol extract was mixed with 1 mL of Folin-Ciocalteu reagent (diluted with H20; 1:1, v/v) and 3 mL of 20% Na_2CO_3 and vortexed. The absorbance of the mixtures was measured at 765 nm after 30 min of incubation in darkness at room temperature. The standard solution of gallic acid (0.5 mg mL^{-1}) dissolved in methanol was used for preparing a calibration curve (0.0–0.10 mg mL^{-1}); its coefficient of variation (r^2) was 0.99. All TP concentrations have been expressed in Gallic Acid Equivalents (GAE)/L of milk.

DPPH scavenging activity

The 2,2-diphényl 1-picrylhydrazyle inhibition (DPPH) was estimated directly into samples without any extraction according to the method described by Alyaqoubi, *et al.* [10]. The DPPH solution was prepared by 40 dissolving mg of DPPH in 100 ml methanol. By mixing 100 mL of the 1 mmol.L DPPH prepared solution with 100 mL methanol. An absorbance of 1 unit was obtained at 517 nm wavelength. Briefly, 0.1 mL milk was mixed with 1 mL freshly prepared 1 mmol.L DPPH solution. The mixture was incubated in the dark for 30 min. A spectrophotometer determined the absorbance at 517 nm. The percentage of DPPH scavenging activity was calculated as follows:

DPPH scavenging activity (%) = [(A blank – A sample) / A blank] × 100,

where A is the absorbance.

FRAP in milk

The ferric-reducing ability of plasma (FRAP) in milk was determined using the method described by Benzie and Strain [11]. The FRAP solution was prepared by mixing 300 $mmol.l^{-1}$ acetate buffer pH 3.6, 10 $mmol.l^{-1}$ TPTZ (2,4,6-tripyridyl-s-triazine) and 20 $mmol.l^{-1}$ $FeCl_3$ in a ratio of 10:1:1. Then, 2 mL of FRAP reagent was mixed with 200 µl of milk extract. This mixture was vortex mixed and incubated in an oven at 37°C for 30 min. The absorbance was then measured at 595 nm. A calibration curve was prepared using an aqueous solution of $FeSO_4$ (0.1–1 mmol/L); FRAP was expressed as mmol $FeSO_4$/L of milk.

Supplementary Material S7

Summaries, ratios and indexes of the fatty acids

The fatty acids profile of milk were used to calculate FA summaries, indexes and ratios.

Short-chain fatty acids (SCFA) = C4:0 + C6:0 + C8:0 + C10:0,

Medium-chain fatty acids (MCFA) = C11:0 + C12:0 + C13:0 + C14:0 + C14:1,

Long-chain fatty acids (LCFA) = C15:0 + C15:1 + C16:0 + C16:1 + C17:0 + C17:1 + C18:0 + 9t-C18:1 + C18:1n-9 + 6t-C18:2 + C18:2n-6 + C18:3n-6 + C18:3n-3 + C20:0 + C20:1 + C20:2 + C20:3n-6 + C20:3n-3 + C20:4n-6+ C21:0 + C22:1n-9 + C22:0 + C22:2 + C22:5n-3 + C22:6n-3 + C23:0 + C24:0 + C24:1,

Saturated fatty acids (SFA) = C4:0 + C6:0 + C8:0 + C10:0 + C11:0 + C12:0 + C13:0 + C14:0 + C15:0+ C16:0 + C17:0 + C18:0 + C20:0 + C21:0 + C23:0 + C22:0 + C24:0,

Mono-unsaturated fatty acids (MUFA) = C14:1 + C15:1 + C16:1 + C17:1 +9t-C18:1 + C18:1n-9 + C20:1 + C22:1n-9 + C24:1,

Polyunsaturated fatty acids (PUFA) = 6t-C18:2 + C18:2n-6 + C18:3n-6 + C18:3n-3 + C20:2 + C20:3n-6+ C20:3n-3 + C20:4n-6 + C22:2 + C22:5n-3 + C22:6n-3,

Desirable fatty acids (DFA) = C18:0 + PUFA + MUFA,

EPA + DHA = C22:6n-3 + 20:5n-3,

n-3 = C18:3n-3 + C20:3n-3 + C20:5n-3 + C22:6n-3,

n-6 = 6t-C18:2 + C18:2n-6 + C18:3n-6 + C20:3n-6 + C20:4n-6,

n-9 = 9t-C18:1 + C18:1n-9 + C22:1n-9,

Atherogenicity index (AI) = (C12:0 + (4 x C14:0) + C16:0)/(MUFA + PUFA),

Thrombogenic index (TI) = (C14:0 + C16:0 + C18:0)/(0.5 x MUFA + 0.5 x n-6PUFA + 3 x n-3PUFA + n-3PUFA/n-6PUFA),

Δ9C14 = (C14:1)/(C14:1 + C14:0),

Δ9C16 = (C16:1)/(C16:1 + C16:0),

Δ9C18 = (C18:1)/(C18:1 + C18:0),

References

1. Aufrère, J.; Michalet-Doreau, B. In Vivo Digestibility and Prediction of Digestibility of Some By-Products. In Proceedings of the Boucque ChV (ed.), Agriculture: Feeding Value of By-Products and Their Use by Beef Cattle. Proceedings of an EEC Seminar, Melle Gontrode; Commission of the European Communities: Brussels, September 26 1983; pp. 25–33.

2. Aufrère, J.; Cartailler, D. Mise Au Point d'une Méthode de Laboratoire de Prévision de La Dégradabilité Des Protéines Alimentaires Des Aliments Concentrés Dans Le Rumen. *Annales de Zootechnie* **1988**, *37*, 233–254.

3. Makkar, H.P.S.; Blummel, M.; Borowy, N.K.; Becker, K. Gravimetric Determination of Tannins and Their Correlations with Chemical and Protein Precipitation Methods. *J Sci Food Agric* **1993**, *61*, 161–165, doi:https://doi.org/10.1002/jsfa.2740610205.

4. Porter, L.J.; Hrstich, L.N.; Chan, B.G. The Conversion of Procyanidins and Prodelphinidins to Cyanidin and Delphinidin. *Phytochemistry* **1985**, *25*, 223–230, doi:https://doi.org/10.1016/S0031-9422(00)94533-3.

5. O'Fallon, J.; Busboom, J.; Nelson, M.; Gaskins, C.T. A Direct Method for Fatty Acid Methyl Ester Synthesis: Application to Wet Meat Tissues, Oils, and Feedstuffs. *J Anim Sci* **2007**, *85*, 1511–1521.

6. Barbano, D.M.; Clark, J.L.; Dunham, C.E. Comparison of Babcock and Ether Extraction Methods for Determination of Fat content of Milk: Collaborative Study. *AOAC* **1988**, *71*, 898–914, doi:https://doi.org/10.1093/jaoac/71.5.898.

7. AOAC (Association of Official Analytical Chemists). *Official Methods of Analysis*; AOAC International, Ed.; 15th ed.; Arlington, 1990;

8. El Otmani, S.; Chebli, Y.; Chentouf, M.; Hornick, J.-L.; Cabaraux, J.-F. Effects of Olive Cake and Cactus Cladodes as Alternative Feed Resources on Goat Milk Production and Quality. *Agriculture* **2021**, *11*, 3, doi:https://doi.org/10.3390/agriculture11010003.

9. Vázquez, C.; Rojas, M.; Ramírez, C.; Chávez-Servína, J.; García-Gasca, T.; Ferriz Martínez, R.; García, O.; Rosado, J.; López-Sabater, C.; Castellote, A.; et al. Total Phenolic Compounds in Milk from Different Species. Design of an Extraction Technique for Quantification Using the Folin–Ciocalteu Method. *Food Chem* **2015**, *176*, 480–486, doi:10.1016/j.foodchem.2014.12.050.

10. Alyaqoubi, S.; Abdullah, A.; Samudi, M.; Abdullah, N.; Radhi Addai, Z.; Al-ghaza, M. Effect of Different Factors on Goat Milk Antioxidant Activity. *Int J Chemtech Res* **2014**, *6*, 3091–3196.

11. Benzie, I.; Strain, J. The Ferric Reducing Ability of Plasma (FRAP) as a Measure of "Antioxidant Power": The FRAP Assay. *Anal Biochem* **1996**, *239*, 70–76, doi:https://doi.org/10.1006/abio.1996.0292.
